# Robots as Mental Health Coaches: A Study of Emotional Responses to Technology-Assisted Stress Management Tasks Using Physiological Signals

**DOI:** 10.3390/s24134032

**Published:** 2024-06-21

**Authors:** Katarzyna Klęczek, Andra Rice, Maryam Alimardani

**Affiliations:** 1 Faculty of Humanities, AGH University of Science and Technology, 30-059 Kraków, Poland; kasia.kleczek@poczta.onet.pl; 2 Department of Computer Science, College of Science, Utah State University, Logan, UT 84322, USA; a02446047@usu.edu; 3 Departement of Computer Science, Faculty of Science, Vrije Universiteit Amsterdam, 1081 HV Amsterdam, The Netherlands

**Keywords:** human–robot interaction, mental health, stress management, emotional response, electroencephalogram (EEG), galvanic skin response (GSR), frontal alpha asymmetry (FAA)

## Abstract

The current study investigated the effectiveness of social robots in facilitating stress management interventions for university students by evaluating their physiological responses. We collected electroencephalogram (EEG) brain activity and Galvanic Skin Responses (GSRs) together with self-reported questionnaires from two groups of students who practiced a deep breathing exercise either with a social robot or a laptop. From GSR signals, we obtained the change in participants’ arousal level throughout the intervention, and from the EEG signals, we extracted the change in their emotional valence using the neurometric of Frontal Alpha Asymmetry (FAA). While subjective perceptions of stress and user experience did not differ significantly between the two groups, the physiological signals revealed differences in their emotional responses as evaluated by the arousal–valence model. The Laptop group tended to show a decrease in arousal level which, in some cases, was accompanied by negative valence indicative of boredom or lack of interest. On the other hand, the Robot group displayed two patterns; some demonstrated a decrease in arousal with positive valence indicative of calmness and relaxation, and others showed an increase in arousal together with positive valence interpreted as excitement. These findings provide interesting insights into the impact of social robots as mental well-being coaches on students’ emotions particularly in the presence of the novelty effect. Additionally, they provide evidence for the efficacy of physiological signals as an objective and reliable measure of user experience in HRI settings.

## 1. Introduction

Stress is a well-known problem among university students, impacting their physical and mental health [1,2]. Several investigations following the COVID-19 pandemic have indicated an increasing prevalence of mental disorders among college students and the fact that the majority do not seek help due to limited financial resources or attitudinal barriers (e.g., embarrassment or preference for self-sufficiency) [3]. This is despite the fact that mental health is an integral part of the Sustainable Development Goals (SDGs) established by the United Nations [4], calling for innovative solutions to promote mental well-being among all groups of people in society. To address this issue, technology-assisted stress management interventions have been proposed as a readily accessible and cost-effective way to reduce stress among students [5,6]. Currently, several smartphone applications [7], chatbots [8], and virtual reality and online games [9] exist that offer guided meditation and deep breathing mindfulness practices.

However, recent human–robot interaction (HRI) studies propose the usage of social robots as a more effective technology for the implementation of stress management interventions [10,11,12,13]. This is because social robots are embodied agents capable of simulating face-to-face social interactions and humanlike behavior. Therefore, compared to other technologies, such as mobile apps and chatbots, social robots can provide a more immersive and personalized experience that can generate more comfort and emotional relief [14,15]. Additionally, they can consistently deliver standardized tasks, reduce stigma, and provide complementary therapy in mental health care, which can be crucial for patients suffering from chronic stress [14]. Thus, robots can be employed as social companions that enhance the effectiveness of technology-mediated therapeutic experiences through meaningful engagement, motivation, and emotional support [14,15].

However, with the growing integration of social robots in mental health interventions, questions about their effectiveness in reducing stress remain unanswered [10,16,17]. This is because past studies have mainly relied on self-reported measures from participants in an uncontrolled experimental setup. For instance, in the study of Jeong et al. [12], a Jibo robot was placed in dormitory rooms on a college campus to deliver daily positive psychology sessions to students. The study collected self-reported questionnaires before and after the intervention, which, depending on the subject, took between 6 and 45 days, and reported an overall improvement in the well-being of students. However, no control condition was considered in this study to isolate the role of the robot in this intervention.

In another study by Spitale et al. [13], two groups of participants used either a QTrobot or a Misty robot which delivered positive psychology exercises in the workplace for a duration of 4 weeks (one exercise per week). The results indicated that the participants who interacted with the smaller and less humanoid Misty robot perceived it more positively than the group who interacted with the QTrobot probably due to the higher expectations they had. While the study provides valuable insights for the design of robotic mental well-being coaches for deployment in real-world environments, it does not provide information regarding their effectiveness in changing participants’ mental states as only perceptions of the robot’s form were collected.

To counteract this issue, the field of HRI can borrow inspiration from affective computing research [18,19,20], in which various physiological signals such as heart rate, skin conductance, respiration rate, and brain activity are used as objective measures of stress levels and emotional responses [20,21,22]. Unlike self-reports that can only be collected once the interaction with the robot is over, physiological signals are time series data that can be recorded with high temporal resolution during the interaction [19]. Additionally, they are less prone to individual biases as they capture unconscious or automatic responses that participants might not express or even be aware of. Therefore, they provide a more accurate and reliable evaluation of the user’s emotional experience that can be used to validate their self-reported perceptions [10,17].

Therefore, this study aimed to evaluate the impact of a robot-guided stress management task on university students by comparing the robot to a control technology such as a laptop. Unlike previous research that mostly relied on self-reported measures from participants, this study took the novel approach of employing neurophysiological signals that gave us an understanding of the change in participants’ emotional experience during the stress management intervention. Therefore, in addition to self-reported questionnaires, this study collected Galvanic Skin Responses (GSRs) and electroencephalogram (EEG) brain activity from two groups of participants who practiced a deep breathing exercise either instructed by a Pepper robot or with the use of a laptop computer. From the GSR signals, participants’ arousal level was extracted and from the EEG signals, the valence of their emotions was extracted (see Background Section for an explanation about the arousal–valence model of emotions). The hypothesis of this research was that the physiological response to the stress management task would be different depending on the technology that was used, with the social robot being more effective in reducing the arousal level and inducing positive emotional valence.

## 2. Background

Affective computing refers to an interdisciplinary field of research that aims to develop systems that can recognize and respond to human emotions [23]. The theories and methods emerging from this field have tremendous potential to improve the quality of HRI by endowing the robots with the ability to recognize human emotions and appropriately respond to them [18,20,23]. This is especially important in application domains such as healthcare, in which understanding a user’s emotional states can help create personalized and more effective HRI [24].

**Figure 1:**
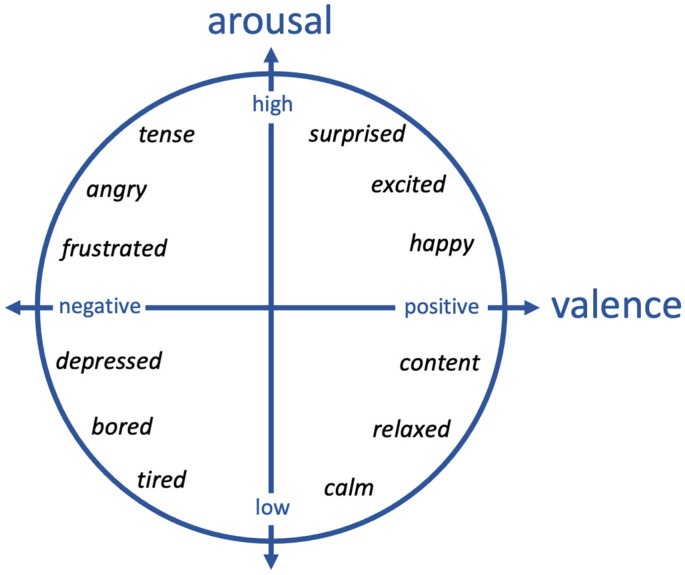
The 2D arousal–valence model of emotions, adapted from [25].

The valence-arousal model [25] is a widely known framework in affective computing research for measuring emotions. In this model, emotions are represented in a two-dimensional space (Figure 1): arousal refers to the intensity of an emotion (how excited or calm the person is) and valence refers to the positive or negative nature of that emotion. Stress is an unpleasant emotion that is generally associated with negative valence and high arousal levels. On the contrary, when a person is relaxed, their arousal level is low, and they are expected to experience a pleasant emotion of positive valence. By extracting the valence and arousal level of participants during a stress management task, it is therefore possible to assess the effectiveness of the intervention in changing the user’s emotional states.

One reliable way to assess arousal from physiological signals is through Galvanic Skin Responses (GSRs). GSRs, also known as electrodermal activity or skin conductance, are obtained by continuously measuring the electrical changes in the skin of the palm or fingers caused by sweat gland activity in response to emotional arousal [26]. Although the usage of GSRs for arousal detection and stress monitoring has been well documented in the affective computing literature [21,26,27] and biofeedback research for stress management [28], very few studies have employed it in HRI settings. For instance, Jerčić et al. [29] applied GSRs, together with a heart rate sensor, to a human–robot collaboration scenario to measure the impact of physiological arousal on people’s performance in a decision-making game task when they collaborated with a robot vs. another human. In another study [30], GSRs were employed in a child–robot interaction to recognize the affective responses of the child to the robot and evaluate their overall experience. These studies, although limited in number, demonstrate the efficacy of GSR sensors in reliably capturing physiological responses during interaction with social robots.

On the other hand, emotional valence can be evaluated by measuring changes in EEG brain activity using the well-known neurometric of Frontal Alpha Asymmetry (FAA) [18,31,32,33]. FAA refers to the lateralization (change between the right and left hemispheres) of the alpha-band brain activity in the frontal brain region. Several neuroscientific studies have shown that a greater alpha activity in the left frontal lobe indicates approach-related emotions associated with positive valence, whereas a greater activity in the right frontal lobe indicates withdrawal responses associated with negative valence [34,35]. The usage of FAA, as a marker of affective states, is becoming popular in the fields of human–computer interaction [36] and consumer psychology [37] for evaluation of user experience and satisfaction. However, in the field of HRI, such research remains scarce. An example is the study of Guo et al. [38], who investigated the impact of a humanoid robot’s emotional behaviors (such as joy, fear, neutral, sadness, or anger) on participants’ emotional responses as quantified by EEG brain signals. They found that participants generated higher Frontal Alpha Asymmetry when they watched the robot’s joy behavior and lower Frontal Alpha Asymmetry when the robot presented sadness behavior.

The current study aimed to address the above-mentioned gap in the literature by exploring the use of physiological sensors for emotion recognition in HRI. By utilizing both GSR and EEG signals, this work presents a novel approach for the detection of valence and arousal in individuals who engaged in a technology-mediated stress management intervention.

## 3. Materials and Methods

### 3.1. Participants

A total of 44 participants were recruited for this experiment and were randomly assigned to one of the experimental conditions. From this number, 8 were removed from the analysis due to noisy physiological signals or technical problems during data collection. Thus, data from 36 participants (20 Females, 16 males, *M*_age_ = 21.3 and *SD*_age_ = 3.71) were included in this study. Among the remaining 36 participants, 18 were in the control group and 18 were in the experimental group. All participants were university students who received course credit in exchange for their participation. The study was approved by the Research Ethics and Data Management Committee of Tilburg School of Humanities and Digital Sciences. All participants received information about the study and provided informed consent prior to participation.

### 3.2. Experimental Design

The experiment consisted of two main parts: (i) a stress induction task, to bring participants to a comparable level of stress [28], and (ii) a stress management task, in which participants performed a breathing exercise with the help of technology. The study employed a between-subject design. This design was chosen to prevent the impact of repeated exposure to the same tasks on participants’ self-reports, as well as physiological responses. The stress induction task was identical for both groups; however, the stress management task differed per group in that the control group used laptop technology that provided written instructions on the screen (Laptop group) and the experimental group interacted with a social robot that provided vocal instructions and gestures (Robot group). Therefore, the main difference between the groups was the technology that was used during the task.

### 3.3. Instruments and Measurements

For the experimental group, a Pepper robot (Softbank Robotics) was used to facilitate the breathing exercise using verbal instructions and gestures, whereas for the control group, a laptop (Acer Nitro 5) was used.

#### 3.3.1. Physiological Measurements

**Figure 2:**
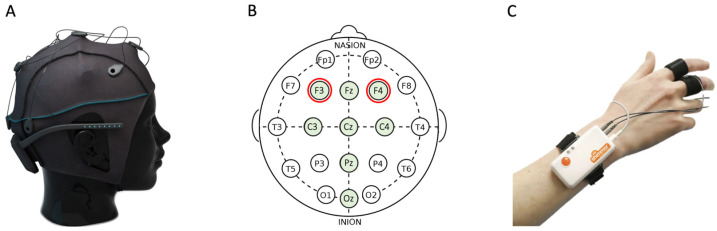
Physiological measurements during the stress management task. (**A**) Brain signals were recorded using a Unicorn Hybrid Black EEG cap. (**B**) The EEG signals were collected from 8 electrodes (shown in green). However, in this study, only electrodes in the frontal region (F3 and F4 marked with red circle) were used for computation of emotional valence. (**C**) Galvanic Skin Responses (GSRs) were recorded using a Shimmer3 GSR+ sensor that was placed on the wrist and fingers of the participants’ non-dominant hand.

EEG brain signals were collected using a Unicorn Hybrid Black (g.tec Medical Engineering, Austria) at a sampling rate of 250 Hz. The Unicorn Hybrid Black consists of a non-invasive wireless EEG cap with 8 electrodes that is worn on the head (Figure 2A). The EEG electrodes were arranged according to the 10–20 international system, as shown in Figure 2B (i.e., green electrodes including F3, Fz, F4, C3, Cz, C4, Pz, and Oz). Before the experiment started, the experimenter placed the electrode cap and applied conductive gel to lower the electrode–scalp impedance. The impedance check was conducted using the Unicorn Suite software. During the recording, the signals were bandpass filtered between 1 and 30 Hz to reduce the noise and the experimenter monitored the quality of signals. In the current study, only signals collected from the frontal brain regions (electrodes F3 and F4 marked with red circles in Figure 2B) were used to extract emotional valence from the brain activity.

Additionally, GSR (sweat) responses were recorded using the Shimmer3 GSR+ (Shimmer Sensing, Ireland) worn on the wrist and fingers (Figure 2C). Shimmer3 GSR+ includes a wireless unit and two dry finger electrodes that are fixed on the non-dominant hand using adhesive strips. The data were collected using the Shimmer ConsensysPRO Software at a sampling rate of 124 Hz.

#### 3.3.2. The Montreal Imaging Stress Task (MIST)

**Figure 3:**
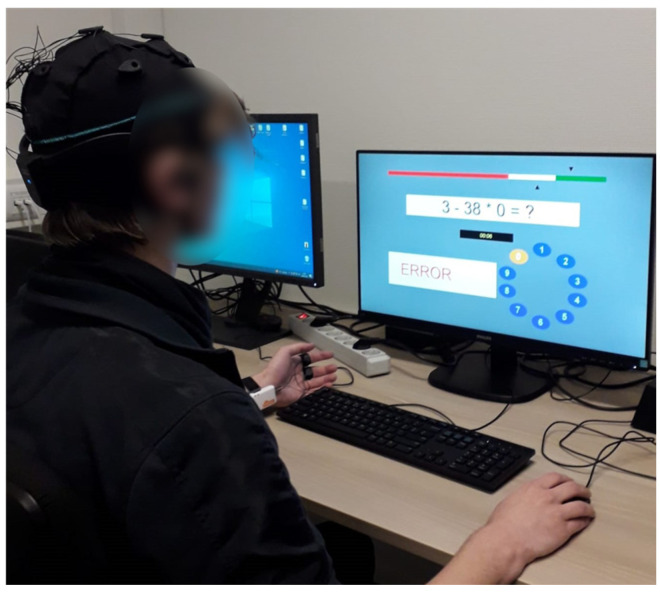
A participant completing the MIST stress induction task.

Before conducting the stress management task with either technologies, participants first engaged in the MIST stress-inducing task proposed by Dedovic et al. [39], which required them to mentally solve arithmetic problems under time pressure. This task aimed to increase participants’ stress levels before the main task to a comparably similar level [28]. The task was conducted on a desktop in the lab using an external application called Inquisit Lab1 [40] (Figure 3). The original test consists of a training trial, a rest period, and five test trials. During the test trials, the participant’s performance is displayed on a performance bar, and the time for each question is displayed; those two factors are the main visual differences between the training and testing trials. Aside from that that, the testing trials also provide less time to answer, and the difficulty level of questions is relatively higher. In this experiment, one example of training and three test trials were used.

#### 3.3.3. The Stress Management Task

**Figure 4:**
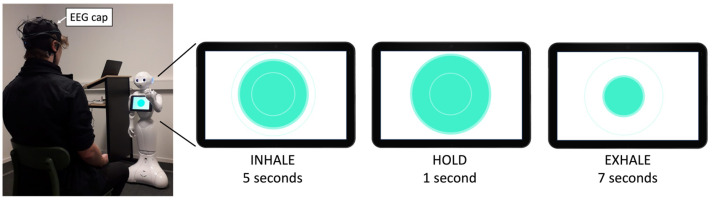
Overview of the stress management task being mediated by a Pepper robot. A video of an expanding and shrinking circle was displayed on the robot’s tablet to guide the participant with the timing of the breathing exercise.

The stress management intervention consisted of a breathing exercise, called “deep and slow breathing”, which has been previously proven effective in reducing stress and slowing down heart rate measures [41,42]. Following the study of De Couck et al. [42], we designed a similar breathing exercise in which participants repeated 5 s inhalations and 7 s exhalations with 1 s holds in between for a total duration of 4 min. To help participants with the timing of the exercise, an instructional video was prepared as a visual aid that was either displayed on the laptop screen (for the Laptop group) or on Pepper’s tablet (for the Robot group) (see Figure 4). The video displayed a circle that slowly expanded to indicate “inhale” and then shrank in size to instruct “exhale” timing.

Participants received explanations about the task and circle movement at the beginning of the breathing exercise. The instructions were either displayed on the laptop screen or told by the robot using an artificial voice. In the Robot condition, the instructions were accompanied by the robot’s arm movements (going upward for inhale and downward for exhale). Additionally, words of encouragement (e.g., “Very good, you are doing amazing”, “Good job”, and “Only 30 s left”) were provided throughout the exercise, either via text (for the Laptop group) or verbally spoken by Pepper (for the Robot group).

#### 3.3.4. Questionnaires

*Affinity for Technology Interaction (ATI).* To ensure that participants in both groups did not have significantly different technological backgrounds, the Affinity for Technology (ATI) scale [43] was administered before the experiment started. The ATI includes 9 items that measure participants’ general comfort and tendency to interact with technology.

*Perceived Stress Levels*. The Perceived Stress Questionnaire (PSQ), originally introduced by Shahid et al. [44], was used to gauge the participants’ subjective level of stress before and after the stress management task. The original scale was adapted from 1 to 4 to a 5-point Likert scale; since the PSQ was collected twice per participant (once before and once after the stress management intervention), it was deemed that the scale adjustment would not impact the overall reliability and validity of the questionnaire.

*Acceptance and Use of Technology*. To determine participants’ impressions of the particular technology they used in the stress management task and their tendency to adopt it in the future, the Unified Theory of Attitude and Use of Technology (UTAUT) questionnaire [45] was administered at the end of the experiment. The UTAUT model is commonly employed in HRI research to evaluate the acceptance of social robots relative to other technologies. For the purpose of this study, we selected seven constructs: “Effort Expectancy”, “Performance Expectancy”, “Perceived Enjoyment”, “Satisfaction”, “Trust”, “Perceived Risk”, and “Behavioral Intention”. For each group, the items of UTAUT were adapted to address the technology that participants interacted with and were rated on a 5-point Likert scale.

### 3.4. Experimental Procedure

**Figure 5:**
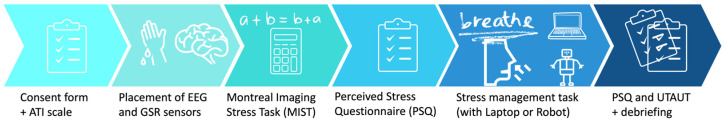
Experimental procedure.

The experiment was conducted in a controlled lab environment and took about 50 min per participant. The general procedure of the study is illustrated in Figure 5. Upon obtaining consent from the participants, the experiment started with the initial questionnaires including demographics and the ATI scale. Then, the experimenter prepared the participant for physiological measurements by placing the EEG and GSR electrodes, which took approximately 15 min. Next, the participant engaged in the MIST stress-inducing task that lasted 5 to 10 min. This was followed by the PSQ questionnaire (5 min), the stress management task delivered either by the robot or laptop (5 min) and the PSQ questionnaire for the second time. Finally, the participant filled out the UTAUT questionnaire and was debriefed (15 min).

### 3.5. Data Processing

#### 3.5.1. EEG Signals and Frontal Alpha Asymmetry (FAA)

The EEG signals were first preprocessed in EEGLAB software (version 2023.0) [46] to remove the noise in the data. First, the “Clean_rawdata” plugin with the default settings was used to remove and reconstruct the data. If channels were removed during this process, electrodes were interpolated. Next, the data were decomposed using the Independent Component Analysis (ICA) implemented in EEGLAB’s pop_runica() function. The selected ICA algorithm was “infomax runica.m” with the default parameters (“extended”, 1). The resulting components were automatically classified using the “ICLabel” plugin in EEGLAB. Components were then inspected using the topographic maps, and those labeled as Eye or Other were rejected, with the “Other” category being rejected if it contained less than 40% brain data.

Once the EEG signals were cleaned, electrodes F3 and F4 were selected for computation of the alpha-band power and consequently the FAA neurometric. The alpha power was calculated by applying the Fast Fourier Transform (FFT) and taking the average power within the specific frequency range of 8–12 Hz. This was conducted in Python (version 3.11) using the NumPy package. Then, using Equation (1), as suggested by Vincent et al. [47], FAA was calculated by subtracting the natural logarithm of the frontal alpha power in the left hemisphere from that of the right side. Positive values of FAA reflect stronger alpha-band activity in the left frontal region of the brain, which represents positive emotional processing, whereas negative values indicate stronger activation of the right frontal regions, indicative of negative emotional processing.

(1) $$FAA=ln({Alpha}_{F3}/{Alpha}_{F4})$$

Since physiological signals entail characteristics that are inherently different across individuals, it is important to normalize the data and look at the relative changes in signal features for each participant rather than the absolute values. Therefore, for each participant, we first computed the FAA in the initial 10 s of the interaction (when the breathing exercise had not started yet) and subtracted it from the FAA calculated in the rest of the recording when the participant engaged in the stress management task (Equation (2)). This yielded a baseline-corrected FAA_score for each participant. A positive FAA_score is suggestive of enhanced positive emotions as compared to the baseline.

(2) $$FAA_{score}=FAA_{task}-FAA_{baseline}$$

#### 3.5.2. GSR Signal and Arousal Level

GSR data were preprocessed using the PyEDA [48]. The cleaned data were then averaged per participant. Similar to FAA, for each participant, the change in their arousal level from the baseline to the stress management task was obtained using Equation (3). Here, a positive GSR_score indicated an increase in arousal levels compared to the baseline.

(3) $${GSR}_{score}={GSR}_{task}-{GSR}_{baseline}$$

#### 3.5.3. Questionnaire Data

The ATI scores were obtained by transforming participants’ answers into numerical form (Strongly agree = 6, Strongly disagree = 1) and then summing all item values. For three items (3, 6, and 8), the values were reverse-coded as the item implied negative sentiment [43].

The PSQ responses were aggregated per subject into one index indicating the stress level before the stress management task (PSQ_before) and after it (PSQ_after). The aggregation of the responses was conducted according to the formula proposed by the original authors in [44]. Since we were interested in the change in perceived stress level, for every participant we acquired one PSQ_score according to Equation (4). A more negative value of this index indicates more stress relief that the participant was able to achieve after the stress management task.

(4) $$PSQ_{score}=PSQ_{after}-PSQ_{before}$$

Finally, the outcome of the UTAUT survey was analyzed by averaging the obtained scores per construct. The mean scores were then compared between the two groups.

### 3.6. Evaluation and Statistical Analysis

#### 3.6.1. Comparison of Robot and Laptop Groups

First, the ATI scores were statistically compared between the two groups to ensure that participants’ background affinity with technology was not a factor impacting the interaction outcomes. Next, to evaluate the effectiveness of the technology that was used in the stress management task in reducing participants’ stress, both subjectively and physiologically, we statistically compared the PSQ_scores, FAA_scores, and GSR_scores between the Robot and Laptop groups. Finally, participants’ perception of the technology they used in the stress management task was evaluated by statistically comparing the mean scores obtained from the seven UTAUT constructs between the two groups. For each analysis, we first checked the normality of the data and equality of variance using the Shapiro–Wilk test and Levene’s test, respectively. Depending on the results, appropriate statistical tests were selected to compare the groups.

#### 3.6.2. Empirical Reconstruction of the 2D Arousal–Valence Model of Emotions

The GSR_scores and FAA_scores were utilized to construct an empirically derived representation of the arousal–valence model of emotions (as seen in Figure 1). Each participant was represented by a data point, with the GSR score as the arousal and the FAA score as the valence of their emotional response during the stress management task. To determine the distinctiveness between the two clusters formed by the Robot and Laptop groups, a Support Vector Machine (SVM) was trained to make the binary classification of the group label. Machine learning algorithms such as SVMs are particularly effective in scenarios where the data are dimensional and not linearly separable, and hence traditional statistical tests are not applicable. This analysis was chosen post hoc once the dissociable pattern between the two clusters of physiological responses was observed. The SVM model (from the scikit-learn Python library) was trained with the 80% of the data to predict the technology used for the remaining 20% data. The mean accuracy in a 10-fold cross validation was then obtained to determine to what extent the Robot and Laptop technologies generated distinct emotional responses.

#### 3.6.3. Relationship between Perceived Stress Level and Physiological Measures

A multiple linear regression analysis using a 6-fold cross validation was conducted to examine if physiological changes during the stress management task (i.e., GSR_score and FAA_score) could predict the change in self-reported stress perception (i.e., PSQ_scores). The test was conducted for each group separately.

## 4. Results

The results of the ATI scale showed no significant differences in technological affinity of the Robot group (*M* = 33.32, *SD* = 5.80) compared to the Laptop group (*M* = 35.26, *SD* = 7.79; *t*(34) = 0.97, *p* = 0.34), indicating a comparable background in interacting with technology. Additionally, to ensure that both groups reached a similar level of stress after the MIST task, the participants’ perceived stress before the stress management task (PSQ_before) was statistically compared between the two groups. The data were normally distributed and therefore an independent sample *t*-test was conducted. The results confirmed no significant difference between the two groups with respect to their stress levels before administration of the stress management task (*t*(31.72)= 1.06, *p* = 0.30).

### 4.1. Impact of Technology on Perceived Stress and Physiological Responses

#### 4.1.1. Comparison of PSQ_scores

**Figure 6:**
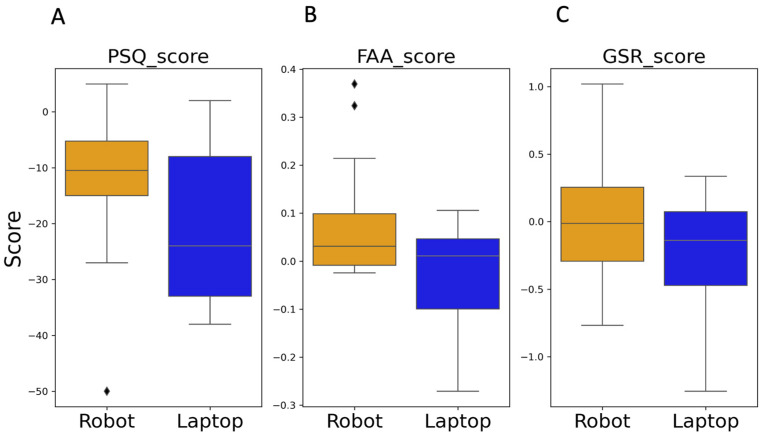
Comparison of the two Robot and Laptop groups in terms of (**A**) the change in their perceived stress (PSQ), (**B**) change in emotional valence (FAA), and (**C**) change in arousal level (GSR).

Since assumptions of normality and variance were met, an independent sample t-test was conducted to compare the PSQ_scores between the Robot and Laptop groups. The test showed no significant differences between groups (*t*(36) = −1.64, *p* = 0.11), suggesting that the technology used for facilitation of the stress management task did not significantly impact participants’ perception of reduced stress (see Figure 6A).

#### 4.1.2. Comparison of FAA_scores

The Mann–Whitney test was used to compare the change in FAA values between the groups, which yielded a non-significant result (*U* = 215.0, *p* = 0.096), although there was a noticeable trend, as shown in Figure 6B, with the Robot group showing generally higher values of FAA_score (which were mostly in the positive range) compared to the Laptop group.

#### 4.1.3. Comparison of GSR_scores

Next, the GSR scores were compared using the Mann–Whitney U test, which revealed a non-significant difference between the Robot and Laptop groups (U = 167.0, *p* = 0.71). In both groups, there were cases of positive GSR_score, indicating an increase in arousal level during the stress management task compared to the baseline (Figure 6C).

### 4.2. The Arousal–Valence Model of Emotions

**Figure 7:**
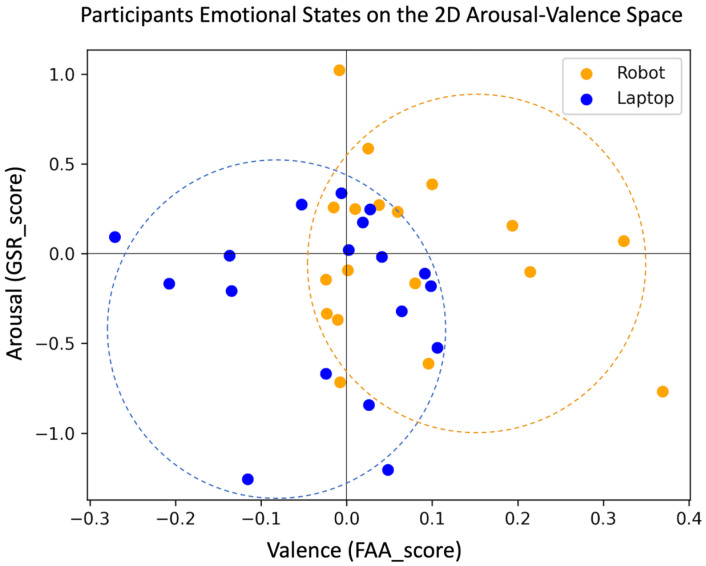
Empirical reconstruction of the 2D arousal–valence model of emotions using participants’ physiological signals (i.e., GSR and FAA features).

Figure 7 displays an empirical representation of the emotional states of participants in each group during the stress management task as quantified by the FAA and GSR scores. The primary objective of this visualization was to emulate the arousal–valence model, elucidating the emotional responses to the Laptop- or Robot-mediated stress management task. As can be seen in Figure 7, two distinct clusters of responses emerged, with the Laptop group presenting reduced arousal accompanied with rather low positive valence or negative valence changes and the Robot group presenting mostly positive valence changes, either with increased arousal or decreased arousal.

To ascertain the difference between the two clusters of data points, we employed an SVM, which is suitable for binary classification and is robust to limited sample size. Using a predictive modeling approach enabled us to go beyond basic statistical testing on either FAA_scores or GSR_scores and show that the two features collectively provide sufficient discriminability between the two groups for supervised classification. The SVM (c = 100, ‘gamma’ = 0.01, kernel = Gaussian radial basis kernel) trained with the GSR_score and FAA_score features achieved a mean accuracy of 0.62 in predicting a participant’s group based on their valence and arousal responses. This outcome underscores the premise that the emotional states induced in participants following the stress management task could be dissociated by a machine learning classifier beyond the chance level (i.e., 50%) and hence differed between the Robot and Laptop conditions.

### 4.3. User Perception of Technology

**Figure 8:**
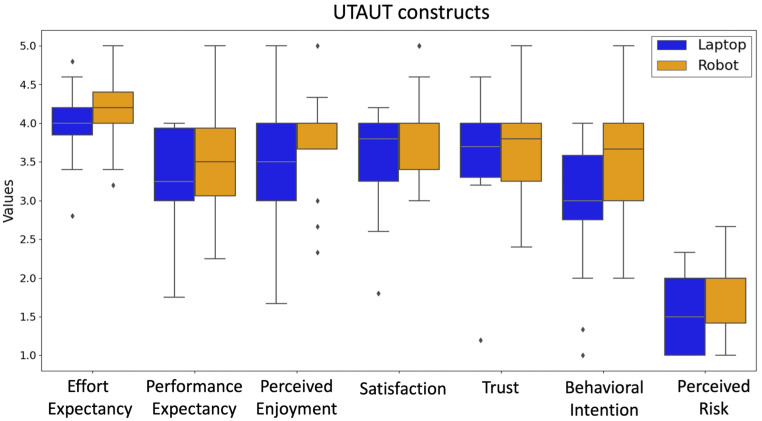
Comparison of the Robot and Laptop groups with respect to their self-reported experience with the technology they used during the stress management task.

Participants’ perception of the Robot and Laptop technology was assessed by calculating the mean scores of each UTAUT construct (Figure 8). Following the outcome of the Shapiro–Wilk test, an independent *t*-test was used to compare the scores of the Effort Expectancy construct and Mann–Whitney Wilcoxon tests were used to compare the remaining constructs between the two groups. The results indicated non-significant differences between groups for all constructs. A trend was seen for Behavioral Intention, showing slightly higher scores reported by the Robot group (*Mdn* = 3.66) than the Laptop group (*Mdn* = 3.0, *W* = 217.5, *p* = 0.07).

### 4.4. Relationship between Perceived Level of Stress and Physiological Responses

A multiple linear regression analysis was conducted to explore the relationship between the subjective and physiological measures of stress in each group. The analysis yielded an insignificant model for the Robot group (*F*(2,11) = 0.08, *p* = 0.92, *R*^2^ = 0.014), as well as the Laptop group (*F*(2,11) = 0.14, *p* = 0.874, *R*^2^ = 0.024). This implies that for all participants, the changes in their GSR and FAA values were not predictive of their self-reported perceived stress.

## 5. Discussion

The current study aimed to test the efficacy of social robots as mental well-being coaches to be employed in stress management interventions. To address the limitations in the past literature concerning uncontrolled experimental design and lack of objective measurements, we compared two groups of participants who engaged in a deep breathing exercise either mediated by a Pepper robot or by a laptop. Additionally, we evaluated the effectiveness of each technology in reducing stress by collecting physiological signals such as GSRs and EEG brain activity, which is a novel approach in HRI research for objective measurement of the users’ experience [10,17,18,19].

The results did not reveal any significant differences between the two groups with respect to their impressions of the technology or self-reported stress levels; however, we observed a notable difference in the way emotional responses emerged in each group of participants. The Laptop group mostly showed a combination of arousal and valence changes that were associated with the third quadrant of the arousal–valence model of emotions (see Figure 1), suggesting emotional states close to boredom or tiredness. On the other hand, the emotional responses from the Robot group were mostly reflected in the first or fourth quadrant of this model, which could be interpreted as either excitement or relaxation, respectively.

Two major implications can be drawn from these findings. Firstly, where *post-interaction* self-reports did not show any differences between conditions, physiological measures collected *during the intervention* provided insights into the variation in emotional responses that users experienced. This corroborates past research on the benefit of wearable technology and affective computing methods in human–computer interaction research [19,27,49], as physiological signals can capture aspects of emotional responses that are more temporally accurate and are beyond respondents’ conscious control or bias [49,50]. Such sensors can play a complementary role in socially assistive HRI by providing valuable insights into unconscious reactions that govern a user’s behavior [30].

Secondly, the physiological responses of the participants in the Robot group seemed to point toward two different emotional experiences that were not originally hypothesized; whereas some could achieve a reduced arousal and positive valence change associated with a relaxed and calm state, others experienced an increase in arousal that was accompanied with positive valence, indicating an experience of excitement or enthusiasm [25]. This unexpected divergence of responses to the robot can be explained in the context of the novelty effect, which was not captured by the self-reported questionnaires. In fact, in experiments with a between-subjects design, where participants cannot employ comparative judgment between conditions, subjective questionnaires may not effectively capture the differences between conditions due to inter-rater variability. This might also explain why PSQ_scores could not be predicted by EEG and GSR measures. These all hint at the importance of physiological measures as a complementary tool in HRI studies [19,49].

While we saw a difference in the way participants’ emotional responses emerged on the 2D arousal and valence space, the difference along each axis of arousal (i.e., GSR values) and valence (i.e., FAA values) was not significantly different between the groups. Previously, Xu et al. [33] conducted a controlled study with a group of students who underwent a 10-week positive psychology intervention and investigated the change in participants’ frontal alpha EEG asymmetry before and after the intervention. In line with the subjective reports of reduced stress, the study observed a significant increase in FAA values in the experimental group compared to the control group, which highlighted the impact of the intervention on neurophysiological responses. Unlike Xu et al. [33], the current study conducted the stress management task only once. Additionally, to isolate the effect of robot interaction, the breathing exercise was conducted by both groups, with the only difference being the technology that was used in delivering the exercise. Therefore, to more robustly examine the long-term effect of robot mediation on participants’ valence and arousal responses, it is necessary to conduct longitudinal studies that investigate user perception and physiology over time [51]. This will enable future research to not only examine inter-individual differences in the adoption and continued use of robots as mental well-being coaches but also evaluate intra-individual differences in showing incremental behavior and physiological changes once the novelty effect wears off [52].

A limitation of the current study is the single interaction that participants had with the technology and the small sample size that was recruited for a between-subjects experimental design. Conducting experiments with neurophysiological sensors, particularly EEG, requires a considerable amount of time and resources to prepare participants [19]. In this experiment, two experimenters simultaneously placed the EEG cap and wrist sensor and checked the impedance and quality of signals before the recording sessions could be started. Initially, we collected data from 44 participants, which is a sizable number for studies employing neurophysiological measurements. However, due to technical difficulties and noise in the signals, we had to discard some participants from the analysis and retain only 18 participants per group. Additionally, due to the limited timeline of the study, it was not possible to invite participants for a repeated measurement over multiple sessions. Future research should try to replicate this study using a longitudinal within-subjects design, for instance by recruiting the same participants on two or more different days to expose and test them in both experimental conditions a repeated number of times. This will not only enable the evaluation of long-term effects but also reduce the impact of individual differences inherent to physiological signals and the novelty effect associated with the use of a new technology on the study outcome [19].

Yet another limitation of the current study was that due to the inclusion of a control technology (i.e., laptop) and the nature of the breathing exercise, the experiment was limited to a short task guided by the robot and thus participants could not fully benefit from a socially interactive experience. An interactive agent can build rapport and a positive relationship with the user that would reinforce the effect of the intervention and increase users’ motivation to continue usage [12]. This interaction could benefit from advanced machine learning algorithms that personalize the robot’s coaching approach based on each individual’s unique needs and emotional responses [53]. Furthermore, future research may employ additional wearables such as respiration and heart rate sensors for multimodal emotional recognition and to verify participants’ performance on the breathing exercise.

Finally, there are several ethical considerations to take into account when using social robots in mental well-being interventions, especially when physiological sensors are integrated. The first is regarding data management and user privacy. Wearable sensors collect sensitive data about an individual’s mental and physical health; hence, it is crucial to ensure that these data are stored securely to protect them from unauthorized access. Next concerns the prolonged impact of robot-mediated interventions, potential dependency, and unforeseen consequences on users’ mental well-being over time. Additionally, employing robots as mental well-being coaches prompts questions about their impact on human autonomy and agency. Users should receive transparent information about the consequences of such technology-mediated interventions and have the option to opt out when they prefer interaction with healthcare professionals. Overall, while social robots provide significant opportunities for future mental health interventions, it is essential to approach them with a strong ethical framework that prioritizes the rights and autonomy of users.

## 6. Conclusions

In conclusion, this study offers valuable insights into the effects of a robot coach on user’s physiological responses during stress management interventions. The study compared a social robot to a laptop in facilitating a breathing exercise for stress reduction. While no significant differences were observed in participants’ perceived level of stress and their general impression of the technology, physiological responses revealed a difference in how emotional responses emerged in each group. The novelty of this work lies in the simultaneous use of EEG and GSR sensors to obtain a holistic understanding of participants’ emotional responses according to the arousal–valence model. A major limitation of the study was the single interaction participants had with the robot, which could have impacted their emotional responses due to the robot’s novelty effect. Further research is needed to validate the effectiveness of robot-mediated mental well-being interventions over time and to understand the relationship between users’ background, impression of the robot, and physiological responses during stress management tasks.

## Data Availability

The raw data supporting the conclusions of this article will be made available by the authors on request.
